# Automatic Clustering and Classification of Coffee Leaf Diseases Based on an Extended Kernel Density Estimation Approach

**DOI:** 10.3390/plants12081603

**Published:** 2023-04-10

**Authors:** Reem Ibrahim Hasan, Suhaila Mohd Yusuf, Mohd Shafry Mohd Rahim, Laith Alzubaidi

**Affiliations:** 1 School of Computing, Faculty of Computing, Universiti Teknologi Malaysia, Skudai 81310, Johor, Malaysia; 2 Al-Nidhal Campus, University of Information Technology & Communications, Baghdad 00964, Iraq; 3 School of Mechanical, Medical and Process Engineering, Queensland University of Technology, Brisbane, QLD 4000, Australia; 4 Centre for Data Science, Queensland University of Technology, Brisbane, QLD 4000, Australia

**Keywords:** kernel density estimation, shared neighbourhood, overlapping diseases, map generation, lesions fragmentation

## Abstract

The current methods of classifying plant disease images are mainly affected by the training phase and the characteristics of the target dataset. Collecting plant samples during different leaf life cycle infection stages is time-consuming. However, these samples may have multiple symptoms that share the same features but with different densities. The manual labelling of such samples demands exhaustive labour work that may contain errors and corrupt the training phase. Furthermore, the labelling and the annotation consider the dominant disease and neglect the minor disease, leading to misclassification. This paper proposes a fully automated leaf disease diagnosis framework that extracts the region of interest based on a modified colour process, according to which syndrome is self-clustered using an extended Gaussian kernel density estimation and the probability of the nearest shared neighbourhood. Each group of symptoms is presented to the classifier independently. The objective is to cluster symptoms using a nonparametric method, decrease the classification error, and reduce the need for a large-scale dataset to train the classifier. To evaluate the efficiency of the proposed framework, coffee leaf datasets were selected to assess the framework performance due to a wide variety of feature demonstrations at different levels of infections. Several kernels with their appropriate bandwidth selector were compared. The best probabilities were achieved by the proposed extended Gaussian kernel, which connects the neighbouring lesions in one symptom cluster, where there is no need for any influencing set that guides toward the correct cluster. Clusters are presented with an equal priority to a ResNet50 classifier, so misclassification is reduced with an accuracy of up to 98%.

## 1. Introduction

Biotic infections can weaken plants and expose them to diseases affecting agricultural production. Signs of these diseases may appear on leaf tissue, and they include noticeable modifications in the colour and shape of the leaves of a plant as it responds to a specific pathogen. Some symptoms look symmetric at different stages of infection, with the possibility of overlapping symptoms appearing on the same leaf. So, the wide variety of symptom characteristics in qualitative and quantitative terms makes it very challenging to collect such samples. It is considered time-consuming and requires experts in the field of agriculture. Thus, more than the collected samples may be needed for training a classifier [1,2,3].

On the other hand, disease detection and identification applications depend on manual data annotation. Symptom datasets are created and labelled manually due to their irregular shapes, which is considered labour intensive and may contain errors or lack of information; hence, only dominant symptoms are annotated and labelled, rather than the other existing minor symptoms. These factors are misleading to the learning process and cause imbalanced class problems. Improving these systems may require automated labelling or region of interest (R.O.I.) segmentation. Therefore, the infected regions in leaf sample images tend to be segmented into individual lesions. However, although this solution has benefits for augmentation and generalisation [2] in the case of rare plants or rare types of overlapping infections, some limitations persist [4,5]. For example, the sensitivity of the automated determination to R.O.I. in images is due to some observations in the background that interfere with leaf boundaries with similar characteristics such as soil and the brown infected regions, and the effect of lights and shadows [6,7,8]. In addition to time complexity, there are concerns about determining the best locations of the centroids and how they are represented within a chosen clustering algorithm, which investigates density or gradient estimation to find differences among observations. These concerns have prompted current attempts to hybridise unsupervised models with specific statistical measures [9,10] or artificial intelligence algorithms to enhance their detection phase.

Concerning the classification phase, studies that applied shallow classifiers confirmed no prior knowledge to determine the best combination of analytical measures and tools needed for lesion determination and disease diagnosis [8,11,12]. Studies applying deep classifiers can eliminate the need for trial-and-error methods until an appropriate approach is found to solve inherent problems with the shallow classifiers. However, deep classifiers still face challenges in implementing the deep layers responsible for the best representation of features [13,14,15]. Features stimulate the classifier and detect symptoms of multiple diseases [16] in cases with mild symptoms [17]. Developing new architectures aims to deploy applications that decrease the computational latency issue with fewer parameters than standard models so that the model can accommodate the target classes taking into consideration an adequate and balanced number of features to train the classifier [2,18,19]. These factors lead to pre- or post-processing to increase accuracy [20,21,22,23], which focuses on the characteristics of the individual lesion in a single leaf [2,18].

In this paper, firstly, we investigate methods that treat R.O.I. independently before classification. Secondly, we explain in detail the proposed framework used in our experiments, which depends mainly on segmenting the R.O.I. and then clustering symptoms that simultaneously appear on a leaf. The performance of these stages is evaluated using a whole leaf dataset that combines rare and varied symptom characteristics, leading us to choose the coffee leaf dataset as a case study [2]. Thirdly, since overlapped infections are rare, collecting such samples in balanced quantities is challenging. This framework is expected to classify individual symptoms better than a whole infected leaf. The classification stage is based on a coffee symptoms dataset. A simple hybrid method is proposed to analyse the behaviour of lesions in the R.O.I. by analysing the distribution of classes. We extract dominant and minor lesions scattered in a leaf sample to validate the idea. Then, we cluster lesions with similar characteristics based on their local densities.

The contributions of this paper are presented as follows:

- The proposed framework is new in the domain of imbalanced data classification, which simultaneously treats major and minor classes by giving them the same priority.
- An effective and quick extraction operation finds symptoms to maintain only regions of infection; the number of classes is validated using the D.B.I.
- A new clustering strategy is adopted to investigate an existing region of infection and that categorises lesions as belonging to single or multiple symptoms.
- The proposed method does not need to predetermine any parameter, which makes it fully automated and flexible. Furthermore, there is no need for an influence dataset to categorise observations.
- The proposed model is simple. Unlike previous models, it allows the self-clustering of overlapped lesions to be classified individually, reducing or preventing misclassification.

## 2. Related Work

Attempts have been made to achieve equality in training datasets to avoid cases where some classes are more dominant than others [24]. Therefore, a balanced dataset is one of the main reasons a classifier can recognise exact features. These problems exist in many applications, such as diagnostic systems in medical fields and health science [25]. A class that appears in enough samples to train a classifier is called a dominant class, while a class that appears in rare instances is called a minority class. The lack of features means occasional classes in samples or minor samples in a dataset. This causes a classifier to ignore the impact of the minority and diagnose the majority. However, a classifier in these cases records high accuracy. If 99% of samples belong to the dominant class, a classifier can correctly diagnose 99% of patients. In this case, a researcher assumes the proposed classification model performs satisfactorily. However, it still neglects the existence of the 1% of the minor class. So, a measure of an imbalanced ratio has been considered [26,27], which estimates whether the classes in a dataset are balanced or not by taking the average of majority classes to minority classes. If the result exceeds one, the set is imbalanced.

There are several types of imbalanced datasets with different imbalanced ratios [28], including minor class samples, overlapping class samples with interfered features, and minor class examples with various features.

Previously proposed methods attempted to achieve balance among observations by resampling classes of a target dataset. Samples were then provided to the learning stage. The goal was to train classifiers with balanced datasets and to prevent misclassification. Some recent methods have been proposed to solve this issue by keeping high-density samples as significant observations, generating similar samples, and avoiding redundancy [26]. However, researchers did not consider that low-density samples may refer to rare or new observations. Up-sampling techniques are based on randomly duplicating minor classes. In this case, overfitting problems can be encountered. At the same time, down-sampling methods can lead to information loss problems due to the random omitting of dominant classes [29]. So, relying on randomisation and generalisation affects the significance of features and their existence in the region of interest (R.O.I.). This is why researchers tend to decrease the error of the local generalisation; to avoid unsatisfactory results, a predetermined distance is chosen based on features’ dimensions within a generalised limit to select the nearest instances to the training dataset [30]. 

Other adaptive methodologies had a different orientation that relied mainly on analysing the R.O.I., such as ensemble methods [31] and data pre-processing or hybridisation methods [32]. In addition to dimensionality reduction techniques such as principal component analysis [33], t-distributed stochastic neighbour embedding [34], canonical correlation analysis [35], and the affinity propagation algorithm [36], the objective is to reduce the dominant class effect and avoid ignorance of any information that refers to rare classes [37]. However, the interference of features makes classes similar, so these methods can only differentiate features if there is clarity in variance among the feature projections of an overlapped R.O.I. Finally, texture analysis methods are strongly sensitive to noise and depend on the clarity of the R.O.I. Any enhancement or structuring method may change the characteristics of the R.O.I. [12,38].

This paper focuses on solving overlapping observation detection problems based on pre-processing methods such as density estimation, clustering, outlier detection, and regression analysis, which are employed to present adequate features for the classification phase.

Clustering-based spatial and density methods include analysing the behaviour and interfered features of extracted regions of interest to differentiate them by determining their gradients [39] and locating the nearest neighbours [40] of observation according to a k-distance. For example, Minkowski distance measures are used to obtain geometric characteristics represented by the centre and radius of a granule to determine its size and location. These characteristics are attained and abstracted from minor and dominant classes [41]. However, the radius threshold value varies as the target dataset varies. 

Sorting the classes according to a hyperplane that depicts relative relationships among points concerning the influence of space surrounding each point can estimate whether it is a target or an outlier [42]. All these methods are strongly affected by many factors: the number of extracted classes and their belonging clusters [43], the local density estimation and the local reachability among connected points, boundaries that separate clusters [44], and local outliers [45]. These parameters vary with the variety of target datasets [46]. So, manually initialising them is considered unhelpful and time-consuming. When the local reachability density factor is a small value, it leads to more confident detection of outlier points. This means it can be affected by the variety of the target dataset and become sensitive to a distant point. However, the local outlier factor is used to measure the degree of outlines of each observation. Nevertheless, it is still sensitive to the spherical distance of nearest neighbours [47,48,49].

One unique solution proposed using adaptive kernel density estimation (KDE) to measure the feature distribution of the observed point and then comparing the resulting probability of that particular point to its nearest neighbours, shared neighbours, and reverse neighbours. It then analysed the fluctuation of that point compared to other points in an R.O.I. by using the average density fluctuation [50,51] to evaluate the outliner indication from that point. This method leads us to propose a new framework that estimates without the need for an influence dataset to depict variety distribution to overcome the problem of predetermining the extent of variability.

## 3. Methodology

The main stages of the proposed framework are conducted as follows. A leaf is subtracted as foreground from a surrounding environment as background [38]. We then extract the whole R.O.I. (combining several lesions), which can contain single or multiple symptoms. It is extracted from the leaf using a modified colour process [38,39]. The number of classes in the R.O.I. is validated using the D.B.I. [43] to ensure no healthy class exists. To determine the existing symptoms, there is a need to fragment lesions in the R.O.I. into sub-images. The local density of each lesion is computed independently so we can find its nearest neighbours according to the kernel probability value that connects it with the other existing lesions. A high probability value refers to the high similarity among lesions. Then symptoms are classified via a ResNet mode. More details are presented to explain the stages of this framework in the following sections.

### 3.1. Dataset

Our methodology uses a dataset with imbalanced instances of various features. This led us to choose the coffee leaf dataset [2], which contains 303 samples of overlapped symptoms and more than 2700 individual symptom samples. In addition to the RoCoLe coffee leaf dataset [52], it includes a single symptom in a leaf with a labelled infection stage, which is used as a reinforcement set to test the proposed clustering phase and to train the classifier in later stages. More details are presented in Table 1.

It is challenging to differentiate the characteristics of coffee leaf symptoms; they can have similar textures, scattered lesions, shapeless lesions, and, at certain stages, more than one colour gradient. For example, Rust and Cercospora have mixed gradients (yellow and brown). Furthermore, they appear in a single leaf at different stages of infection. Obtaining enough samples with such variety to train a classifier is time-consuming and demanding.

### 3.2. Proposed Framework

**Figure 1:**
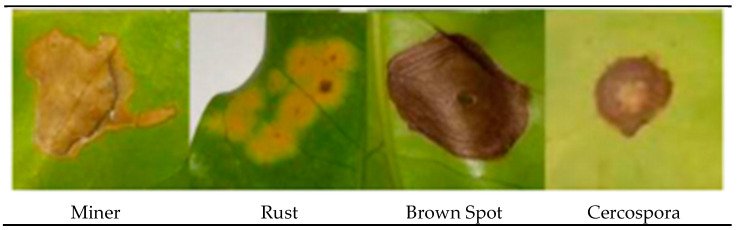
The main symptoms categorisation according to [2].

**Figure 2:**
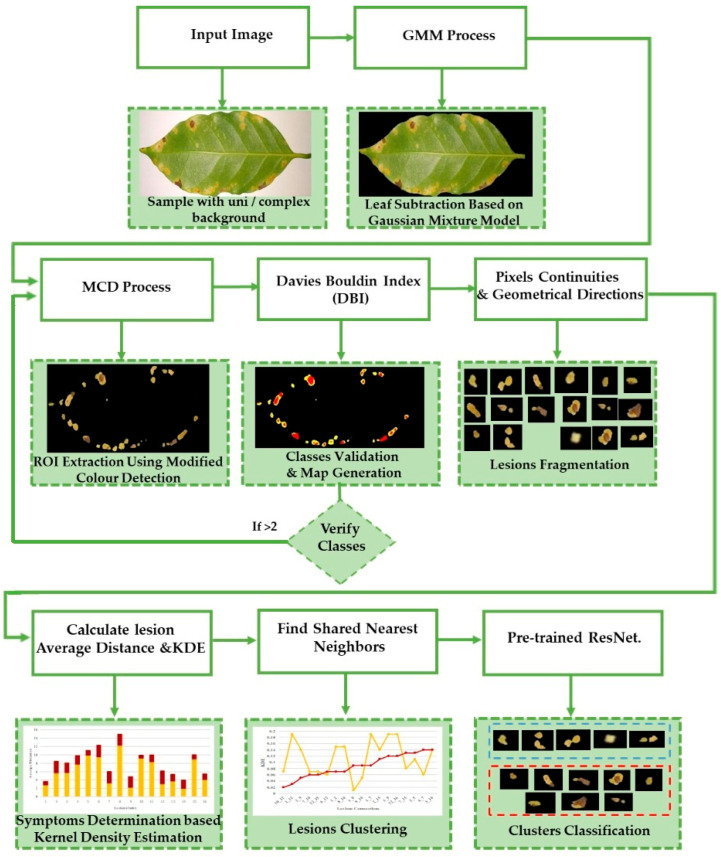
The flowchart explains the primary stages of the proposed framework.

Our method suggests handling sparsity caused by overlapped symptoms with multiple classes/features. Therefore, the existence of multiple classes may refer to the presence of single or various symptoms. In the case of early infection, symptoms appear with one class. At later stages, some symptoms appear with interfering classes. As seen in Figure 1, the Rust sample shows a sign with multiple classes (yellow and brown gradients). These main stages of the framework are presented in Figure 2.

#### 3.2.1. Stage1_ROI Extraction

At this stage, the leaf is estimated to be subtracted from a complex background according to a previously proposed method based on the graph cut and Gaussian mixture model [38]. The injured regions (R.O.I.) are obtained from the leaf by removing the healthy regions as presented in Algorithm 1.
**Algorithm 1: R.O.I. Extraction.****Input**: Coloured image of a leaf sample.**Output**: Two matrices of modified green pixels (M.G.P.) and modified red pixels (M.R.P.).**Step 1**: Process a modified colour-based detection method (M.C.D.) to check leaf pixels in an image. The red and green pixel values (R.P.V. and G.P.V.) are subtracted from the greyscale image value (G.I.V.):(1)Modified red pixels (M.R.P.)= R.P.V.− G.I.V.(2)Modified green pixels (M.G.P.)= G.P.V.− G.I.V.**Step 2**: Keep pixels with yellow and brown gradient only, which are responsible for determining symptoms regions. Equations (3) and (4) validate that: (3)$\mathrm{Red}\;\mathrm{pixel}\;\left(R.P.\right)=\;M.R.P.-\frac{G.P.V.}{2}+\frac{B.P.V.}{2}$(4)$$M.R.P.=\{\begin{array}{c} 0,\;\;\;\;\;\;\mathrm{else}\\ p\left(i,j\right),\;R.P.\left(i,j\right)\geq \;\mathrm{threshold} \end{array}$$

Equation (1) determines pixels with yellow gradients, while Equation (2) specifies pixels with brown colour. However, it is challenging to differentiate yellow from light green in a leaf sample; images are affected by factors such as image-capturing conditions and lighting effects. Furthermore, healthy regions become less bright in the advanced disease life cycle than other leaves with other infection stages. Even the yellow scale varies according to an infection level. Therefore, there is no predefined colourful spectrum to confine green and yellow gradations for all the leaf samples with single/multiple infections.

**Figure 3:**
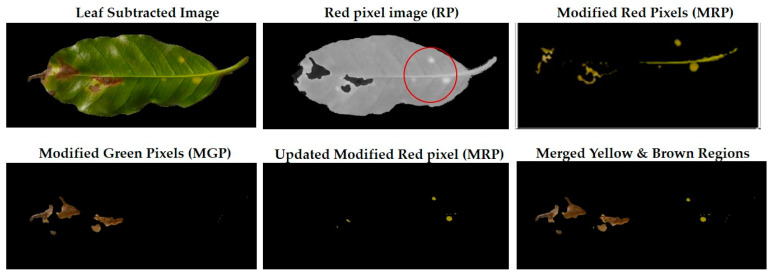
The R.O.I. extraction stage after applying the D.B.I. metric. The red-circled regions contain values higher than the most repeated ones.

Additional equations solve this problem. Equation (3) determines the range of yellow and light green gradients, and Equation (4) is responsible for refining these gradations by estimating the threshold value. The threshold should be larger than the R.P. matrix’s most repeated values. Hence, repeated values represent the healthy regions. Pixels with values larger than the repeated values represent infected regions (generally, yellow gradients are higher than green values). Figure 3 shows an example. In the Modified Red Pixel (M.R.P.) image, the obtained yellow regions contain some light green pixels, representing the regions affected by lighting conditions, where the main vein appeared as part of the R.O.I. There was a need to validate the number of extracted classes to solve this problem.

#### 3.2.2. Stage2_Class Determination and Validation

At this stage, the number of classes is verified using the D.B.I. It is the ratio of the sum that differentiates classes. This method is addressed to validate the number of obtained classes, as presented in Algorithm 2. In this phase, the R.O.I. is represented by the unhealthy regions. According to the target dataset, one class in the R.O.I. refers to one type of infection. In this case, Equation (6) returns a value of one. On the other hand, two classes in the R.O.I. can refer to overlapped symptoms or a single symptom at an advanced stage of infection. In this case, the D.B.I. returns a value of two.
**Algorithm 2:** Class Verification.**Input:** M.G.P. and M.R.P. matrices.**Output:** D.B.I. value to confirm the number of existing classes and ROI_img, the R.O.I. generated map.**Step 1:** $\mathrm{Calculate}\;\mathrm{the}\;\mathrm{centres}\;\mathrm{of}\;\mathrm{classes},\;\mathrm{where}\;C_{i}$$\;\mathrm{is}\;\mathrm{the}\;\mathrm{mean}\;\mathrm{of}\;\mathrm{pixels}\;\mathrm{obtained}\;\mathrm{from}\;\mathrm{Equation}\;(2),\;\mathrm{and}\;C_{j}$ is the mean of pixels obtained from Equation (4).**Step 2:** $\mathrm{Calculate}\;\mathrm{the}\;\mathrm{distances}\;\mathrm{of}\;\mathrm{points}\;\mathrm{to}\;\mathrm{their}\;\mathrm{class}\;\mathrm{centres}\;(W_{i}+{\;W}_{j}$) using the Euclidean distance function:(5)$$\;D=\sqrt{\overset{n,m}{\underset{i,j\;=1}{\sum }}{\;W}_{i}+{\;W}_{j}}$$W_i_ represents the average distance of all points in class C_i_ to their cluster centres, and W_j_ represents the average distance of all points in class C_i_ to the centre of class C_j_.**Step 3**: Compute the D.B.I., where C_ij_ represents the distance between the centres of classes C_i_ and C_j_.(6)$$\mathrm{DBI}\left(k\right)=\frac{1}{k}\sum_{i\;=1}^{k}\max\frac{W_{i}+{\;W}_{j}}{C_{\mathrm{ij}}}$$**Step 4**: Judge the convergence; if DBI ≥ 3 then:Increment the threshold value in Equation (4).Update the M.R.P. matrix.Repeat Steps 1,2,3.         Otherwise: continue to Step 5.**Step 5:** Merge the M.G.P. and M.R.P. matrices to integrate both brown and yellow gradients in one R.O.I. image (ROI_img).

Sometimes, the validation process in Equation (6) may not refer to the optimal number of classes in the R.O.I. due to the appearance of light green pixels (healthy regions). Therefore, the D.B.I. may exceed the value of two, meaning there is a third class with a different ratio that needs to be omitted to reduce the index value. The threshold value in Equation (4) is altered until we obtain an optimal M.R.P. matrix, which is responsible for yellow gradients. In Figure 3, two extracted features are shown in the R.P. image (yellow regions) and the M.G.P. image (brown regions). However, the returned D.B.I. value exceeds two due to the healthy region accompanying the yellow regions. Therefore, the M.R.P. should be altered by changing the threshold value of Equation (4). If we find more than one value higher than the most repeated values and they appear with an equal amount, the M.R.P. matrix is altered several times until the D.B.I. becomes less than or equal to two. Figure 3 shows the results of the updated M.R.P. before and after obtaining the optimal threshold value.

#### 3.2.3. Stage3_Lesion Fragmentation

The main idea of this stage is to locate and fragment all lesions in the regions of interest, as presented in Algorithm 3. Each lesion is kept in a sub-image to independently analyse its characteristics; the number of detected lesions in a leaf is determined by locating the boundaries of each lesion. This depends on pixel intensities, continuities, and directions.

**Figure 4:**
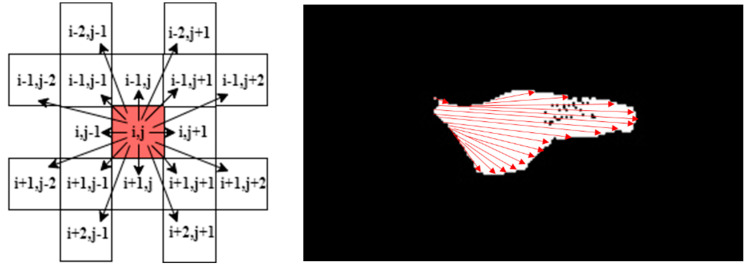
Tracking the continuities in all directions for a lesion; discontinuities with small distances are ignored.

A lesion is a group of connected points. By detecting the first point in a lesion, we keep tracing the connected points until discontinuity occurs in all eight directions, as in Figure 4. This means all points in this lesion are selected to be saved in a sub-image indexed by the number of detected lesions. We then search for a new k lesion until all the lesions in the R.O.I. are visited and determined.
**Algorithm 3:** Lesion Determination and Fragmentation.**Input:** updated ROI_img.**Output:** ROI_generated map, K lesions sub-images.**Step 1:** Unify colours by changing all yellow gradients to (R:255, G:255, B:0) and all brown gradients to (R:255, G:0, B:0).**Step 2**: Detect points of ROI_img and trace continuities of successive pixels in the eight directions:  While i < ROI_img (height) Do:   While j < ROI_img (width) Do:    If ROI_img (i, j) > 0:      At each of the following directions (i, j + x), (i, j − x), (i − y, j − x), (i − y, j),     (i − y, j + x), (i + y, j − x), (i + x, j), (i + y, j + x) assign the value of the     detected point to its correspondent location in sub-image k.     x and y are temporary counters initialised to the location of a current     point; they increment by one to visit the next point, until they obtain a     zero-pixel value, then jump to the next direction.    Else continue searching for a new point until each point in this matrix is    visited once.   End  End**Step 2**: Initialise K according to the number of extracted lesions. **Step 3**: For each lesion in the R.O.I., ensure that each point in that lesion is selected: (7)$$N\left(i,j\right)=\overset{i\;+\;x}{\underset{m\;=\;i\;-\;x}{\sum }}\overset{j\;+\;y}{\underset{n=\;j\;-\;y}{\sum }}D\left(m,n\right)$$where (i, j) is the location of a current point and (x, y) is the location of the farthest point in a lesion dimension. This equation supposes that each point in the map within these dimensions should have a corresponding point in the lesion sub-image. Otherwise, we assign the actual value of that pixel to the lesion sub-image. This step ensures that all the points in that lesion are integrated. Taking into account the noise and holes according to an acceptable spatial distance threshold ϵ compared to the surrounding points within the lesion:(8)$$N\left(L_{k}\right)={\;\{L}_{k}\in \mathrm{ROI}\;|d\left(p_{x,y},p_{i,j}\right)\;\leq \;\epsilon \}$$$\mathrm{where}\;L_{k}$ is a lesion with k index that belongs to an R.O.I. of a leaf sample.

Before fragmenting lesions, a map is generated according to the validated R.O.I. image, as in Figure 2. The objective of developing the map is to create a reference image of locations and feature/class distribution in the R.O.I. This map unifies colour gradients (one colour refers to all the existing yellow gradients, and another refers to brown gradients). In this way, we can quickly determine classes and their distributions in each lesion.

To validate the detected lesions, Equations (8) and (9) check whether all the points have been selected by comparing the current lesion sub-image with the generated map.

#### 3.2.4. Stage4_Symptom Determination and Classification

In this stage, the obtained lesions are analysed for clustering. We addressed an extended KDE to define relations among lesions and presented it in Algorithm 4. The local density of each lesion in a leaf is measured, and then lesions with a similar probability are clustered together. The KDE is applied to nonparametric problems; when observations of a target dataset appear with unlabelled proportions of outlines, the KDE estimates every point that does not belong to a selected bandwidth as an outlier. For our chosen dataset, the R.O.I. combines overlapped diseases. When the minor symptom is considered an outlier, the primary symptom is considered a standard observation. This method is harnessed to estimate lesions with similar probabilities as neighbours are grouped in the same cluster. Lesions related to the dominant symptom (first cluster) are assumed to appear with closer density distributions than those of the minor sign (second cluster), which are considered outliers.
**Algorithm 4:** Symptom Determination and Classification.**Input:** Map_ image.**Output:** Clusters of images.**Step 1**: Initialise K according to the number of extracted lesions. **Step 2**: For each lesion in the R.O.I.:      Calculate the average distance for points characterised as yellow in the      map separately from brown points using the Euclidean distance measure. **Step 3**: Find the nearest neighbours to a current lesion according to the similarity of characteristics based on the adaptive weighted Gaussian kernel function: (9)$$\;\rho \;\left(p_{i}\right)=\overset{n}{\underset{j\;=1}{\sum }}\frac{w_{j}}{h_{j}^{d}}\;K\left(\frac{p_{i}-{\;p}_{j}}{h_{j}}\right)$$(10)$$W_{j\;}=\frac{a\;-\sum_{j\;=1}^{\;n}\mathrm{Euclidean}\left(p_{i,}p_{j}\right)}{a}$$$\mathrm{where}\;p_{i}\;$$\mathrm{is}\;\mathrm{the}\;\mathrm{average}\;\mathrm{distance}\;\mathrm{of}\;a\;\mathrm{current}\;\mathrm{lesion},{\;p}_{j}$$\;\mathrm{is}\;\mathrm{the}\;\mathrm{average}\;\mathrm{distance}\;\mathrm{of}\;\mathrm{the}\;\mathrm{estimated}\;\mathrm{neighbour},\;k\;\mathrm{is}\;\mathrm{the}\;\mathrm{Gaussian}\;\mathrm{kernel}\;\mathrm{and}\;w_{j}$ represents the weight computed by measuring the Euclidean distance between two lesions.(11)$$\;K\left(\frac{p_{i}-{\;p}_{j}}{h_{j}}\right)=\frac{1}{2\pi }\exp\left(\frac{-\|p_{i}-{\;p}_{j}\|{}^{2}}{2\times {\;h}_{j}^{d}}\right)$$The h value is adapted to handle bandwidth estimation and accommodates each spot in a leaf.(12)$$h_{i}=\alpha [d_{k}\_\max\;+{\;d}_{k}\_\min\;+\delta -d_{k}\left(p_{i}\right)]$$$\mathrm{The}\;\mathrm{parameters}\;d_{k}\_\max{\mathrm{and}\;d}_{k}\_\min$ are the maximum and minimum distances of yellow and then brown points for each lesion in a single leaf sample.**Step 4**: Sort the obtained probabilities in ascending order, then arrange lesions into two main groups, where a group represents a symptom; points that share equal or similar probabilities are categorised as neighbours in one group.**Step 5**: Classify each group independently using ResNet50 classifier.

## 4. Results and Analyses

This section discusses the obtained results and compares similar previous studies in the field. All the proposed framework experiments and comparisons are performed via Intel(R) Core(T.M.) i7-4710HQ CPU, 8G memory and the Windows 10 Pro operating system. The Anaconda platform is used with the Python 3.7 programming language.

### 4.1. Parameter Settings

Clustering overlapped symptoms with different rates, including interfered features with no prior knowledge, is considered a nonparametric problem; the parameters have no fixed values. Values change concerning a leaf sample, so parameters would be determined by the number of existing lesions in a leaf and their attributes. An adaptive width and weight are used in Equations (11) and (13) to avoid under-smoothing, over-smoothing, and negative kernels that result from the disparity between the farthest and nearest point in the R.O.I.

The ρ parameter represents the probability of similarity/difference. It is calculated by setting the weight and width among the current and existing spots in a leaf sample until all the k spots are visited. According to the literature [50], the value a is the largest Euclidean distance among points and is used to normalise results. In our proposed method, it is set to the average distances of extracted lesions to avoid negative kernels.

However, some parameters are considered default parameters; the value α is a scaling factor that ensures distance smoothness among lesions. According to the literature, the value of α ranges from 0 to 1, regardless of whether the data is synthetic or real. The value of the δ parameter guarantees that the width will never be zero; δ is recommended to be a small positive value, which we predetermined to be 0.01. However, the value of δ does not change the result but prevents the kernel width from being zero.

### 4.2. Experimental Results

In Table 2, there are eight leaf examples (five examples have different overlapped diseases, and the others have a single type of symptom but with overlapped features and at different infection levels). Two features have been extracted from stage 2 (yellow and brown) in the first leaf example. Nine fragments (lesions) have been obtained from stage 3. Stage 4 presents two lesions; hence, relevant lesions in each group of symptoms share similar probabilities of a specific class (feature).

The density estimation probabilities are measured for all the existing lesions in a leaf. These probabilities are sorted in ascending order, and lesions with close probabilities are sorted as neighbours in the same group, namely, symptom 1. Hence, they share the same characteristic (the highest probabilities). The remaining lesions with lower probabilities are combined with the second group, namely, symptom 2.

**Figure 5:**
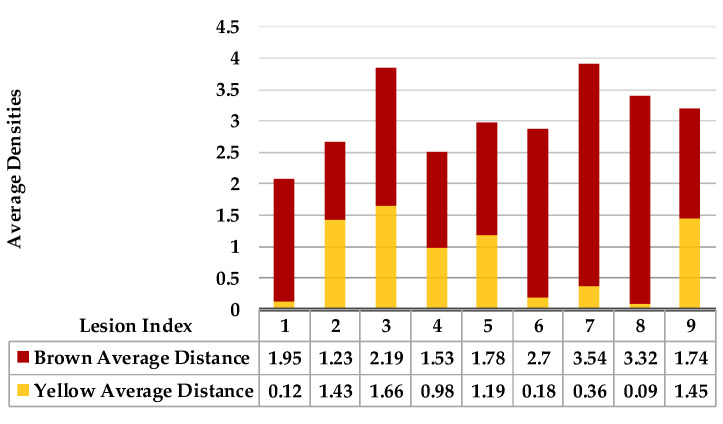
Leaf 1, the difference among lesions’ distances.

**Figure 6:**
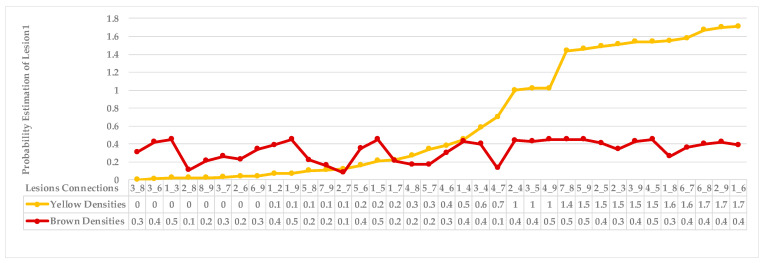
Leaf 1, variety in local density estimation among lesions in a leaf sample.

Figure 5 shows lesion’s average distances of brown and yellow points of leaf 1. Figure 6 shows that the highest ρ value for yellow density (1.7) is obtained between lesion 1 and lesion 6, which means lesion 6 is the nearest neighbour to lesion 1. They are added together in the same cluster {1,6}. Lesions (6_8) share the same probability value; hence, lesion 6 is the common neighbour between lesion 1 and lesion 8. That makes lesion 8 join to the same cluster {1,6,8}, namely, symptom 1. Then, lesion 7 is added to group symptom 1 due to the common neighbour lesion 6, becoming symptom 1 = {1,6,7,8}. The process is continued until all the lesions are sub-grouped. Successively, lesions (2_9), lesions (2_3), and lesions (2_5) are grouped in symptom 2 due to the shared neighbour lesion 2. Finally, lesion 4 is joined to symptom 2 using lesion 5 lesions (4_5). Symptom 2 = {2,3,4,5}.

**Figure 7:**
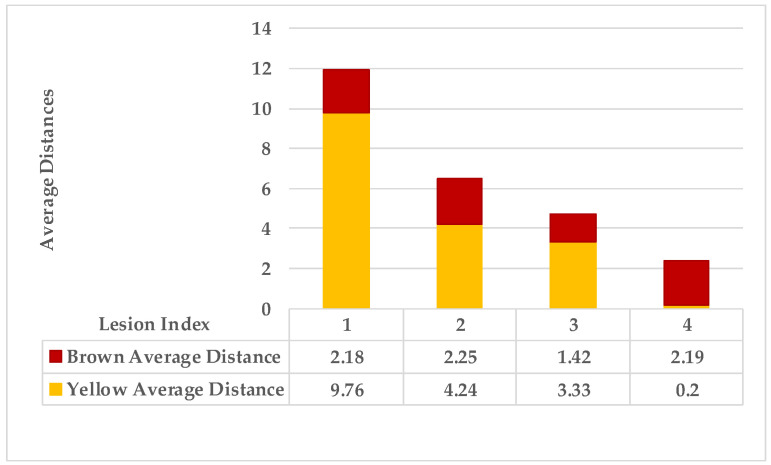
Leaf 2, the difference among lesions’ distances.

**Figure 8:**
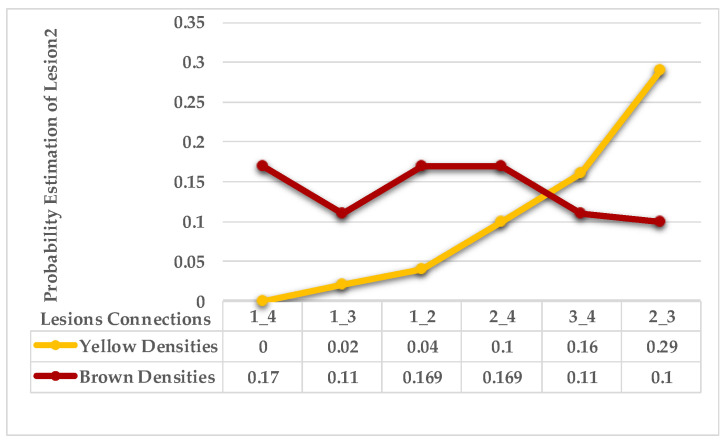
Leaf 2, variety in local density estimation among lesions in a leaf sample.

The second example in Table 2 is Leaf 2, which contains two symptoms and two extracted classes. Figure 7 shows each lesion’s average distances of brown and yellow points. Figure 8 shows that lesions (2_3), lesions (3_4), and lesions (2_4) have a typical neighbourhood, and their average probability of density estimation (ρ value for yellow pixels ≥ 0.12). They are combined in symptom 1 = {2,3,4}. At the same time, lesion 1 is the farthest in this neighbourhood. Due to the low probability values that connect lesion 1 with the lesions {2,3,4}, lesion 1 belongs to group symptom 2.

**Figure 9:**
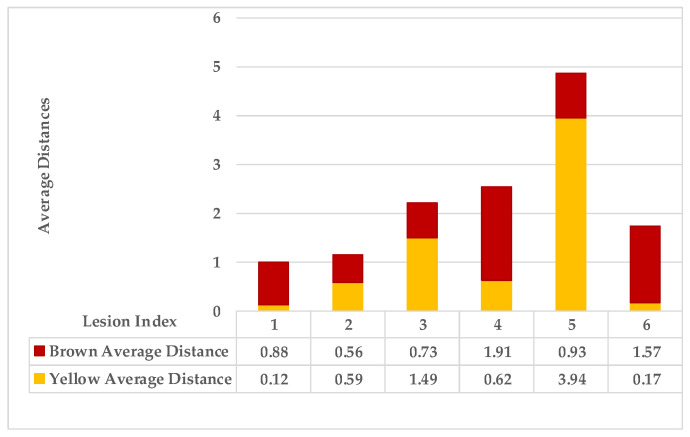
Leaf 3, the difference among lesions’ distances.

**Figure 10:**
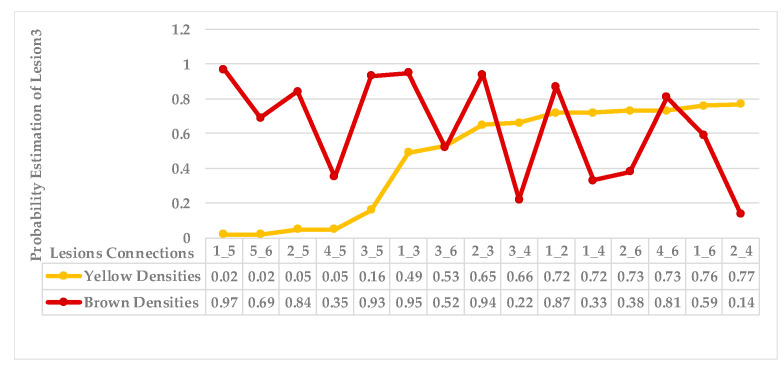
Leaf 3, variety in local density estimation among lesions in a leaf sample.

The third leaf sample has two overlapped symptoms and two features. One of the symptoms appears with one feature (brown gradients only), while the second symptom appears with mixed features (brown and yellow gradients). Figure 9 shows the average distances of all lesions of this leaf. Figure 10 shows that lesion 5 is the farthest one in the group (symptom 2). In contrast, the other lesions have similar probabilities (more significant than the average of the probabilities) and are clustered into the group (symptom 1).

**Figure 11:**
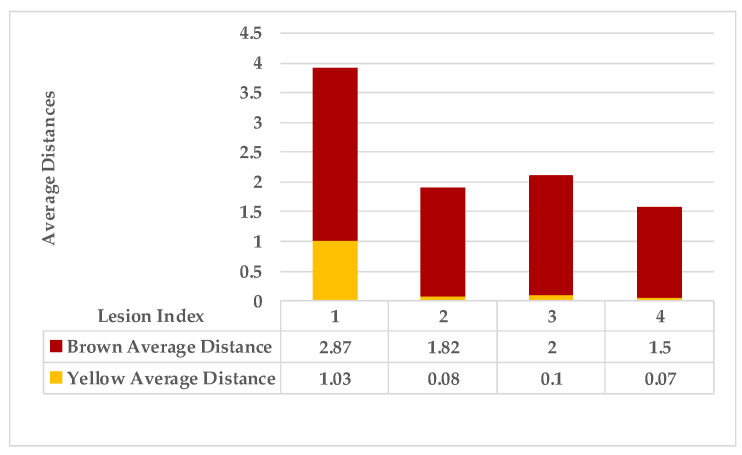
Leaf 4, the difference among lesions’ distances.

**Figure 12:**
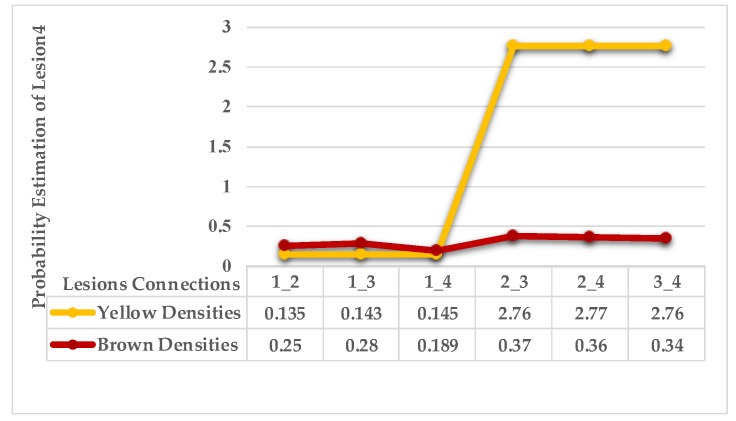
Leaf 4, variety in local density estimation among lesions in a leaf sample.

In the fourth leaf example, a lesion with mixed features is the first observation. In contrast, all the remaining lesions have a single feature (brown gradients). As shown in Figure 11 the average distances of these lesions. Figure 12 shows the probabilities of lesions (3_4), lesions (2_4), and lesions (2_3) are higher than the average of the probabilities. That places lesions {2,3,4} in the same group (symptom 1). In contrast, lesion 1 is the farthest lesion due to its low connectivity estimation to others.

**Figure 13:**
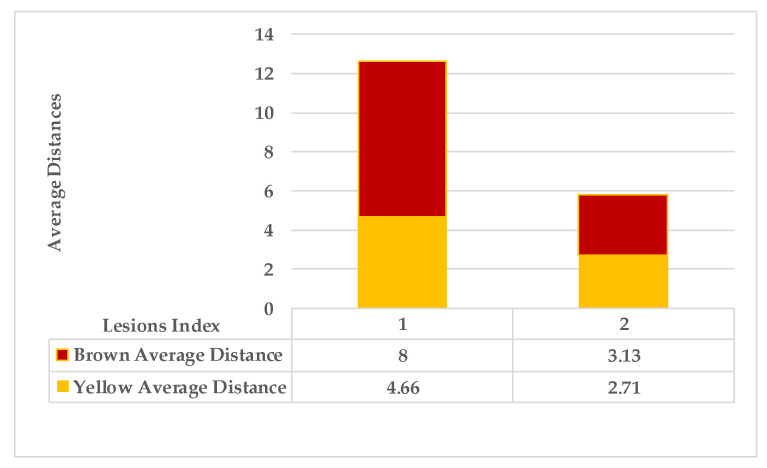
Leaf 5, the difference among lesions’ distances.

**Figure 14:**
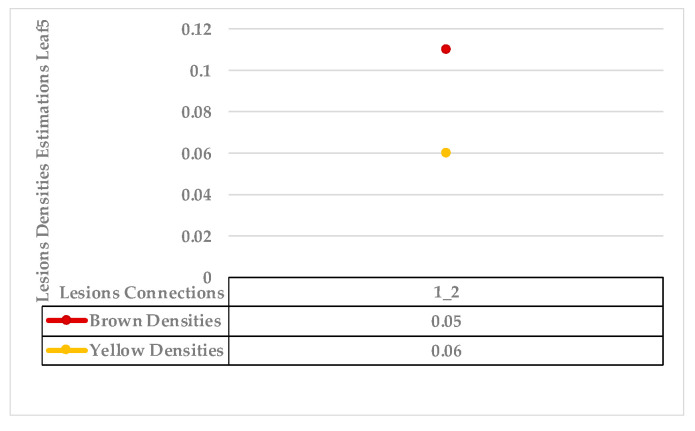
Leaf 5, variety in local density estimation among lesions in a leaf sample.

The fifth example combines only two lesions with mixed features (yellow and brown gradient pixels). Figure 13 shows variance in average distances of both lesions. The obtained probability density estimation of lesion 1 to lesion 2 is very low (ρ for yellow density = 0.06), as shown in Figure 14. There are no other lesions with which to compare. According to the target datasets, the least similarity estimations exceed the value of 0.1, weighting the possibility of two different symptoms.

**Figure 15:**
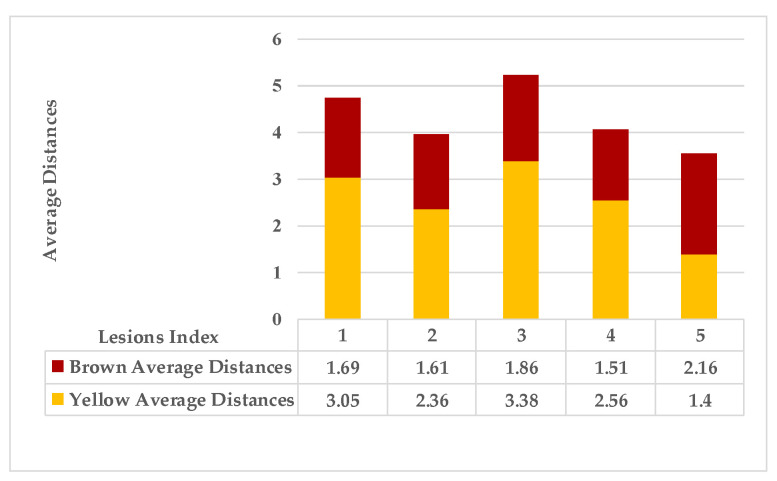
Leaf 6, the difference among lesions’ distances.

**Figure 16:**
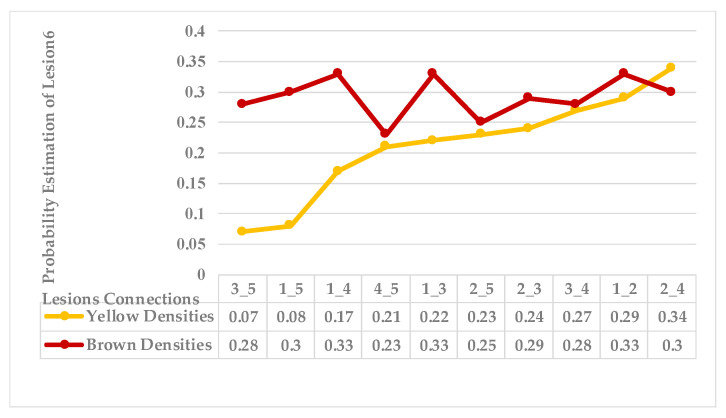
Leaf 6, variety in local density estimation among lesions in a leaf sample.

The sixth leaf sample has several close lesions of mixed features, as shown in the generated map and Figure 15 shows the average distances of these lesions. However, all these lesions belong to a single symptom. According to Figure 16, lesions (2_4), lesions (1_2), and lesions (3_4) have a shared neighbourhood, and the estimations are higher than the average. Therefore, all lesions {1,2,3,4} are grouped before reaching the average value = 0.1.

**Figure 17:**
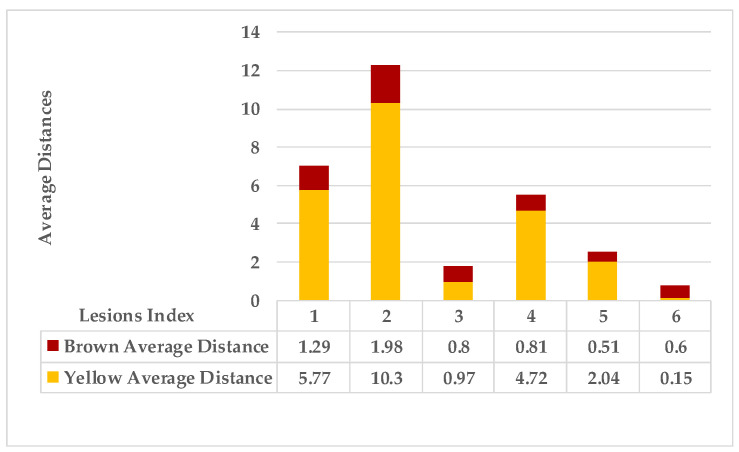
Leaf 7, the difference among lesions’ distances.

**Figure 18:**
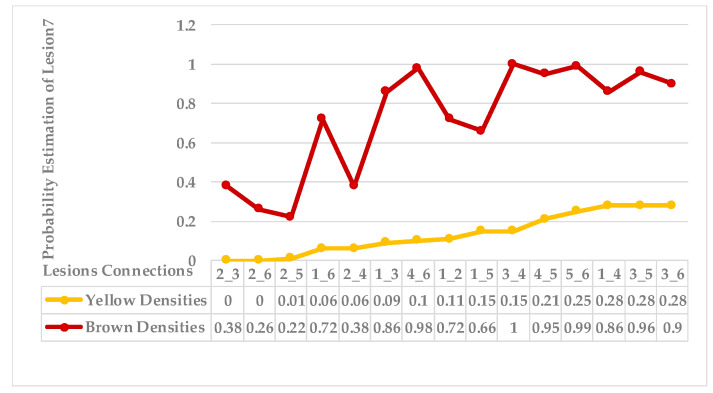
Leaf 7, variety in local density estimation among lesions in a leaf sample.

**Figure 19:**
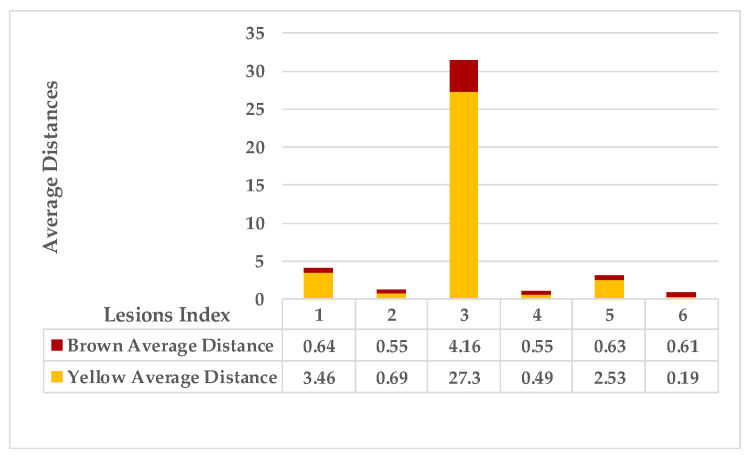
Leaf 8, the difference among lesions’ distances.

**Figure 20:**
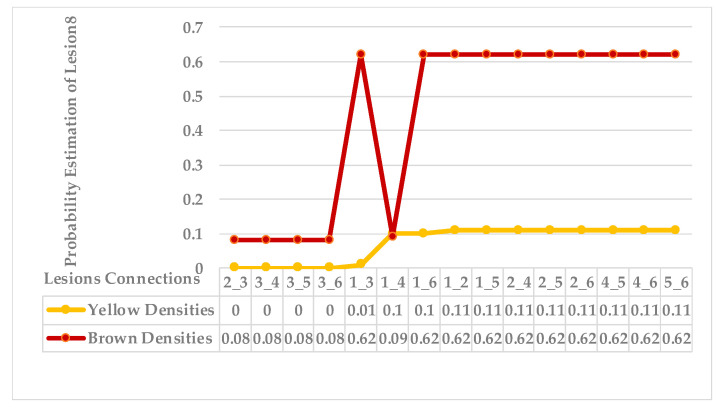
Leaf 8, the difference among lesions’ distances.

The seventh sample has a single advanced infection level with mixed features. All lesions are connected with an average probability estimation of density distribution (ρ value for yellow densities ≥ 0.13) and belong to the same symptom group. The obtained sub-images contained more than one lesion due to their tiny size; however, they are scattered along the leaf but are very close to each other. Results are shown in Figure 17 and Figure 18. Finally, the last leaf has the same characteristics as sample 7, except it has a different level of infection. As shown in Figure 19 and Figure 20.

### 4.3. Classification Results

In the classification phase, Residual Networks (ResNet50) was chosen due to the deep layers in its architecture that increase its efficacy in feature detection.

In Table 3, we compared our method to similar previous methods; the classifiers are trained using the same available coffee leaf datasets where leaves are infected with biotic stresses. They proposed to segment R.O.I. first and then classify the dominant symptom neglecting any minor observations that lead to misclassification.

### 4.4. Extended KDE Analysis

It is challenging to guarantee the distances among lesions of the same symptom due to the overlapped classes; interference causes similar average distances, and taking their total average increases ambiguity, so the generated map image with unified colours is created to measure the average of yellow distance and the average of brown distance separately for each lesion by determining the minimum and maximum distances for both gradients.

As mentioned in Section 2, the basic methods of nearest neighbours depend mainly on the distance threshold and the value of k. In the case of imbalanced datasets, however, these parameters are affected by the dominant symptom (the variety in features’ distributions in the R.O.I.). Therefore, it is difficult to determine the threshold–neighbourhood extent and the border among symptoms. Distances are very close and vary, as shown in Figure 5, Figure 7, Figure 9, Figure 11, Figure 13, Figure 15, Figure 17 and Figure 19. To solve the problem of border symptom separation, Stage 4 gathers lesions according to their average distances and then smooths them using a modified kernel density estimation. The farthest/nearest spots from a current location are selected based on their probabilities of density estimation, as shown in Figure 6, Figure 8, Figure 10, Figure 12, Figure 14, Figure 16, Figure 18 and Figure 20. Points with high-density estimates are grouped as neighbours in one cluster. They are more likely to be similar in their density distribution than lesions of another cluster.

### 4.5. Analytical Comparison with Other Kernels Methods

The chosen density estimation method is compared with the radial bias function kernel (R.B.K.), Adaptive_RBK [48], and Epanechnikov Kernel [56]. Different density bandwidth selectors are tried to reduce the cluster density estimation error.

- RBK:

(13) $$K(X_{i},X_{j})=\exp\left(-\frac{\|X_{i\;}-{\;X}_{j}\|{}^{2}}{2\delta^{2}}\right),$$

where $\|X_{i}-X_{j}\|{}^{2}$ is the Euclidean distance between two lesions. The kernel value varies in the limit (0 and 1). The recommended bandwidth estimator selection (h) is the global alignment kernel [57]:

(14) $$\delta =\;\mathrm{median}\left(\|X_{i}-{\;X}_{j}\|{}^{2}\right)\times \sqrt{N}$$

For an adaptive R.B.K. [48], more parameters are added for the bandwidth selector, to be implemented as follows:

(15) $$\delta =\;\mathrm{median}\left(\|X_{i}-{\;X}_{j}\|{}^{2}\right)\times \frac{\ln K}{\ln N\;\times \beta }$$

where K is the number of lesions in a leaf, β is an iterative parameter, and N is the number of points in a lesion.

- Epanechnikov kernel:

(16) $$K(X_{i},X_{j})=\frac{3}{4h}\left(1-\left(\frac{\|X_{i}-{\;X}_{j}\|{}^{2}}{h^{2}}\right)\right)$$

where the probability [58] of kernel extent is:

(17) $$K\;\mathrm{Epanechnikov}=\{\begin{array}{c} \frac{3}{4}\left(1-{\left|x\right|}^{2}\right)\;\mathrm{if}\;\left|x\right|<1\\ 0\;\;\;\;\;\;\;\;\;\;\;\;\;\;\;\;\;\;\mathrm{elsewhere} \end{array}$$

The recommended bandwidth estimator selection (h) is Scott’s rule of thumb [59]:

(18) $$h\approx 1.06\times \hat{\sigma }n^{\frac{1}{5}}$$

As it is fixed for all the lesions, $\hat{\sigma }$ is the standard deviation of the R.O.I., and n is the number of R.O.I. points.

**Figure 21:**
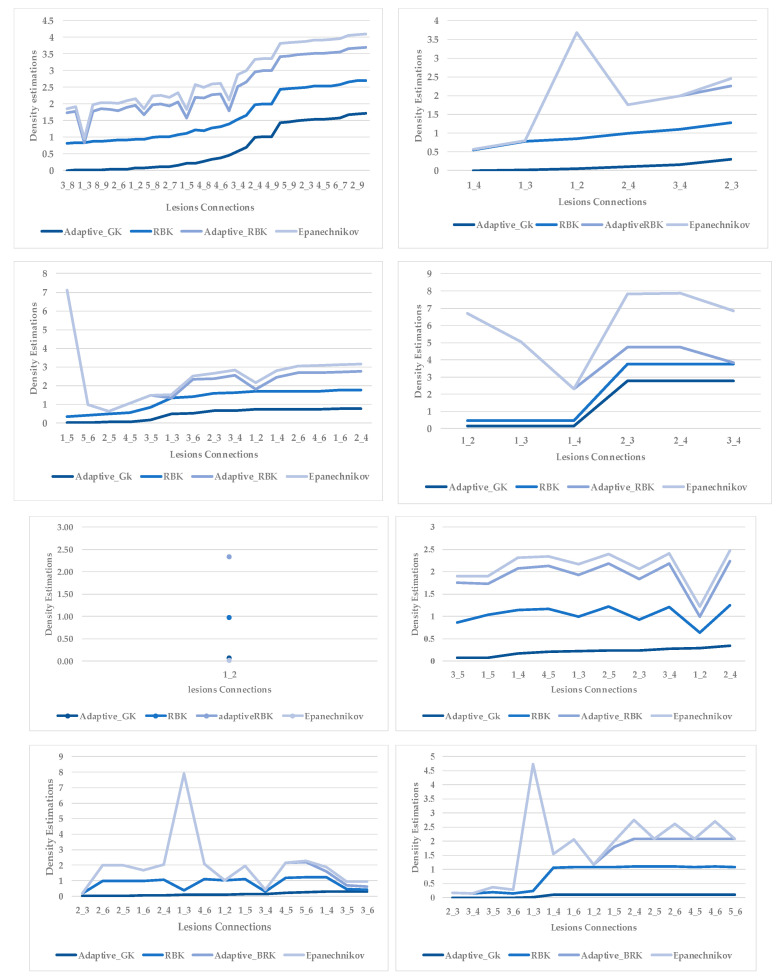
Probability density estimation according to the proposed adaptive Gaussian kernel, radial bias function kernel, adaptive radial bias function kernel, and Epanechnikov kernel methods.

As clarified in Figure 21, the proposed kernels for comparison are applied for the same overlapped cases successively selected in Table 2. The radial basis function kernel has almost the exact probabilities as the adaptive Gaussian kernel. In contrast, the other types of kernels have slight differences. For more detail, the confusion matrix has been chosen to show the best kernel method.

**Figure 22:**
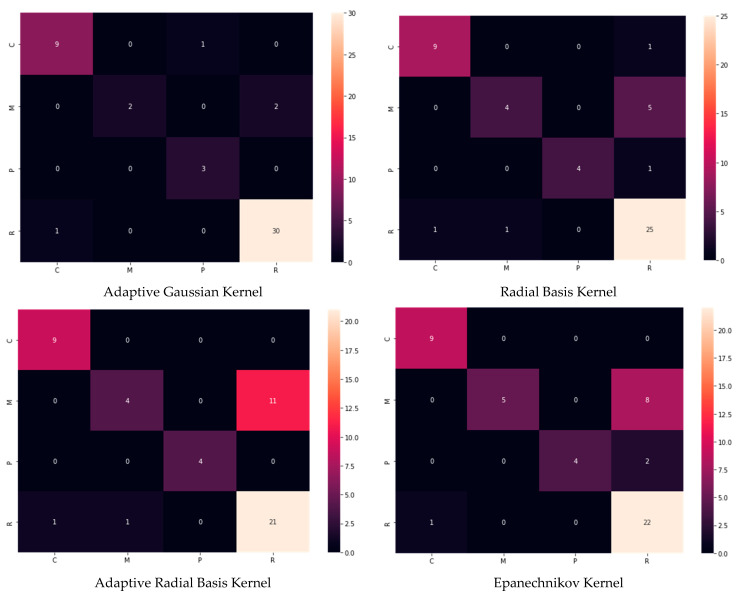
Confusion matrix of the used kernels.

The confusion matrix results shown in Figure 22, where there are some advanced cases of single infection with overlapped features (especially Rust and Cercospora), can be explained as follows. At certain levels of disease, the features look similar, and the kernels suggest the existence of two clusters (the probabilities indicate that there are two symptoms). However, the lesions should be categorised as belonging to a single cluster (single symptom). The adaptive Gaussian kernel shows the minimum categorisation error compared to other kernels.

However, there are other bandwidth estimators, such as Silverman’s rule of thumb, where we obtained ambiguous results. Hence, Scott’s rule of thumb is more suitable for the normal distribution. Furthermore, the balloon estimator needs more parameters to be predefined for each leaf, such as the centre and length of the spherical space for the target cluster, which made it unfavourable to be used in the proposed method.

## 5. Discussion

In this section, we present the current issues that led to the proposed framework of a fully automated diagnosis system for leaf plant diseases, as follows:

- There is a need to extract the R.O.I. method without losing region properties. The modified colour process is proposed to assume that the darkest gradients refer to the brown injured regions and the lightest gradients refer to the yellow injured regions.
- The best analytical technique to analyse variety in syndrome is self-clustering based on an extended Gaussian kernel density estimation method. This method avoids overfitting and over-generalising problems that result from resampling observations to provide a balanced dataset. Furthermore, it avoids over-smoothing that results from undesirable bandwidth selectors. Hence, the bandwidth is adaptive to the R.O.I. of each leaf.
- Most classification models are developed to detect prevalent diseases in a leaf. The solution is proposed to improve the classification of leaf disease diagnosis by making it able to characterise multiple infections in the same leaf by clustering symptoms and then training a classifier using a balanced symptoms dataset. So, each cluster is classified independently, reducing the classification error percentage.

## 6. Conclusions and Future Work

Imbalanced observations are a common challenge in the field of machine learning and data analysis, especially in the context of classification tasks. The coffee leaf dataset is an excellent example of such a scenario, where one or more classes in the dataset are underrepresented compared to the others. This can lead to a bias in the learning process, as the algorithm may tend to favour the majority class over the minority class [60].

It is important to remember that these techniques should not be applied blindly but with a thorough understanding of the dataset and the problem. The choice of technique will depend on the dataset’s specific characteristics and the classification task’s requirements. Emphasising the attributes of the minority class individually through techniques such as resampling, weighting, or a combination of both can help to mitigate the effects of class imbalance and prevent the model from favouring one class over the other.

It was challenging to determine the probabilities of a cluster, but the proposed method proved its efficacy in specifying similarity among related lesions. Moreover, compared to other kernel methods, probability determination was more straightforward. The obtained probability value is either 0 or 1, which means lesions with zero probability belong to the same cluster; otherwise, they belong to the other cluster. However, these kernels failed to categorise cases of single advanced infection, treating lesions as belonging to two different clusters.

The proposed method relies mainly on R.O.I. fragmentation into individual lesions, where each lesion is treated as a point that may belong to one of the existing clusters in a leaf. However, some sporadic cases were found with overlapped symptoms in a single manually fragmented lesion. Therefore, we recommend lesion analysis as an autonomous R.O.I. in future work to avoid this issue.

## Figures and Tables

**Table 1:** R.O.I. datasets.

Dataset	Biotic Stress	No. R.O.I. Images
Coffee dataset	Miner	593
	Rust	991
	Phoma	504
	Cercospora	378
	Healthy	272
	Miners and Phoma	1
	Rust and Phoma	2
	Brown spot and Cercospora	7
	Miners and Cercospora	15
	Miners and Rust	112
	Rust and Cercospora	166
	Total	3041
RoCoLe dataset	Rust	602
	Healthy	300
	Total	902

**Table 2:** Lesions’ neighbourhood-relation-based KDE and similarity in characteristics.

Symptoms	Leaf Image	D.B.I.	Generated Map	No. Detected Lesions	No. Groups
Rust and Cercospora	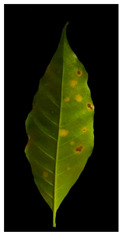	2	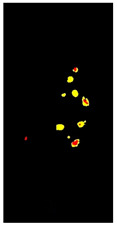	9	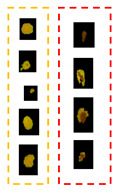
Rust and Phoma	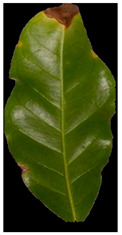	2	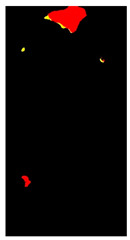	4	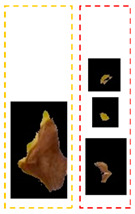
Rust and Miner	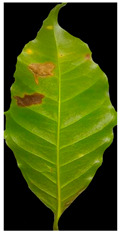	2	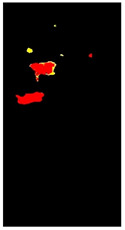	6	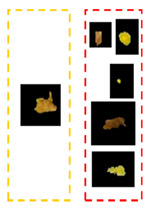
Phoma and Cercospora	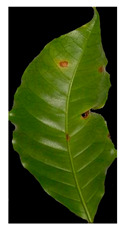	2	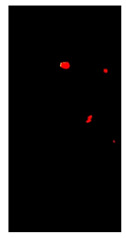	4	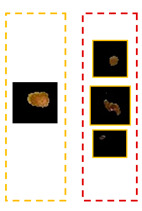
Miner and Cercospora	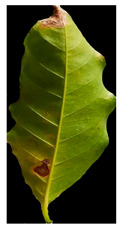	2	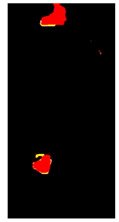	2	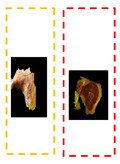
Miner	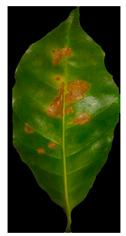	2	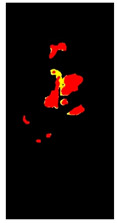	5	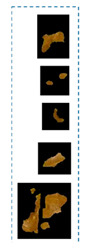
Rust	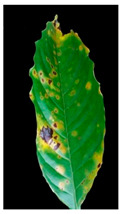	2	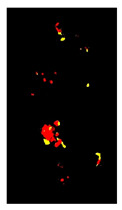	6	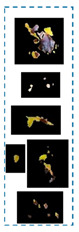
Rust	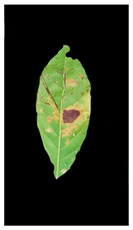	2	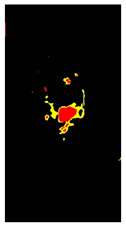	6	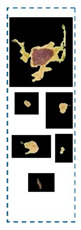

**Table 3:** The obtained results for coffee symptoms classification using different architectures compared to the proposed method.

Model	Accuracy
Multi-task CNN [53]	95.63%
ResNet50 [53]	97.07%
PSPNet + ResNet [54]	94.17%
TripletNet (ResNet50 as Backbone) [55]	95.82%
Extended KDE + ResNet50	98%

## Data Availability

Data supporting this study are available at the following repositories: Krohling, Renato A.; Esgario, Guilherme J. M.; Ventura, José A. (2019), “BRACOL—A Brazilian Arabica Coffee Leaf images dataset to identification and quantification of coffee diseases and pests”, Mendeley Data, V1, doi: 10.17632/yy2k5y8mxg.1; Parraga-Alava, Jorge; Cusme, Kevin; Loor, Angélica; Santander, Esneider (2019), “RoCoLe: A robusta coffee leaf images dataset”, Mendeley Data, V2, doi: 10.17632/c5yvn32dzg.2.
